# Pulse of the Pandemic: Iterative Topic Filtering for Clinical Information Extraction from Social Media

**Published:** 2021-02-13

**Authors:** Julia Wu, Venkatesh Sivaraman, Dheekshita Kumar, Juan M. Banda, David Sontag

**Affiliations:** 1 Dept of Electrical Engineering and Computer Science, Massachusetts Institute of Technology; 2 Human-Computer Interaction Institute, Carnegie Mellon University; 3 Department of Computer Science, Georgia State University

**Keywords:** data mining, social media, information retrieval, topic modeling, clinical concept extraction, public health surveillance

## Abstract

The rapid evolution of the COVID-19 pandemic has underscored the need to quickly disseminate the latest clinical knowledge during a public-health emergency. One surprisingly effective platform for healthcare professionals (HCPs) to share knowledge and experiences from the front lines has been social media (for example, the “#medtwitter” community on Twitter). However, identifying clinically-relevant content in social media without manual labeling is a challenge because of the sheer volume of irrelevant data. We present an unsupervised, iterative approach to mine clinically relevant information from social media data, which begins by heuristically filtering for HCP-authored texts and incorporates topic modeling and concept extraction with MetaMap. This approach identifies granular topics and tweets with high clinical relevance from a set of about 52 million COVID-19-related tweets from January to mid-June 2020. We also show that because the technique does not require manual labeling, it can be used to identify emerging topics on a week-to-week basis. Our method can aid in future public-health emergencies by facilitating knowledge transfer among healthcare workers in a rapidly-changing information environment, and by providing an efficient and unsupervised way of highlighting potential areas for clinical research.

## 1 Introduction

In May 2020, a retrospective study of over 3,000 patients in a major New York healthcare system found that in around 1% of cases, the disease caused by the novel coronavirus (COVID-19) was associated with ischemic stroke [27]. The result, which was initially described in early April in China and Europe [16, 15] and corroborated by other New York studies [13, 21], quickly became part of a larger story on thrombotic complications of COVID-19 [12]. In the weeks leading up to these articles, however, physicians were already discussing a possible association between cerebrovascular accidents and COVID-19 on Twitter, starting with a small number of users affiliated with Boston-area hospitals on March 17. If conversations like these could be surfaced to physicians around the world as they emerge, they could serve as a focal point for new evidence, suggest directions for clinical research, and accelerate progress toward understanding the disease.

In the face of a developing medical situation such as COVID-19, health-care professionals (HCPs) interact with a range of knowledge sources to provide and share up-to-date, accurate clinical information. Guidance released by public health organizations such as the World Health Organization (WHO) and the US Centers for Disease Control and Prevention (CDC) is considered the most reliable, but is relatively slow to change due to its wide impact. These guidelines are backed by published research and case reports, which also take time to be disseminated due to the need for formalization and peer review. To obtain more up-to-the-minute information, HCPs share insights amongst each other through webinars, hospital-specific channels, and chat groups. For example, many initial accounts of Chilblain-like skin lesions on the toes, a now-famous symptom of COVID-19, were circulated on a WhatsApp group from France [17].

Social media platforms have emerged as important areas for sharing clinical information publicly. In particular, Twitter is a popular option because it is already home to a sizeable physician community [22, 19]. HCPs can opt to discuss medical topics under hashtags such as “#medtwitter” and “#epitwitter,” or share experiences tagged as “free open-access medical education” (#FOAMed). The community is considered useful for networking, sharing knowledge, and continuing education [22], although it can pose risks to credibility and privacy [7]. While Twitter would not be appropriate as a primary source of clinical guidance, it has been shown to be a useful complement to more incremental, rigorously evaluated sources [28], and to assist in the dissemination of new information across geographic and cultural boundaries [23].

The task of *finding* clinical information on social media, however, can be challenging. For HCPs looking for advice or anecdotes, it may be difficult to find the most relevant authors unless they are already in the right social circles. Furthermore, because of the high volume of non-expert conversation, the terms that one would expect to find in clinically-meaningful information can also be found in mundane and non-expert posts as well as in myths and misinformation [24]. In the case of COVID-19, even within the posts that mention the pandemic, the global impact on everyday life has essentially put “popularity” at odds with medical usefulness.

### 1.1 Automating clinical relevance filtering

Framed as an information retrieval problem, our task is to extract and cluster clinically relevant social media posts by reputable authors, who form a tiny minority of the general population. Our method is therefore designed to implicitly derive a set of distinguishing characteristics between relevant and irrelevant text, building upon easily-available indicators such as medical vocabularies and user metadata. While we exclusively consider COVID-19 and Twitter data in this study, the problem formulation and approach can be applied to other healthcare topics or social media platforms.

For the purposes of this study, we define information as *clinically relevant* if it is technical in nature and intended to help characterize, prevent, diagnose, or treat the disease under consideration. While some past approaches designate relevance through manual labeling [14, 10], this is difficult to scale due to the time sensitivity of a pandemic and the rapid shifts in conversation topics over time. Therefore, we turn to topic modeling with Latent Dirichlet Allocation (LDA) [5], an unsupervised approach that has also been studied extensively in the literature [11, 8, 29]. However, the level of granularity that has typically been achieved in these approaches is limited by the user’s ability to scan through and interpret topics. Hierarchical topic models may facilitate interpretation by connecting related topics [4, 25], but due to the sheer volume of irrelevant data in social media, we propose that filtering out the undesirable items altogether may be a more robust strategy.

We take an iterative approach to finding the most clinically relevant documents within a dataset. Documents are automatically annotated for clinical concepts using MetaMap [1], which provides an initial approximation of clinical relevance (though prone to false positives [9]). Our method uses topic modeling to associate documents with similar content without supervision, then scores topics based on the relevance of the clinical concepts they contain. The documents are then filtered by their degree of association with the most relevant topics, and the process is repeated. The concept relevance estimates are refined in each iteration, thereby overcoming the noise in concept annotations as the filtering quality improves.

We demonstrate the utility of our method by retrospectively analyzing 1 million automatically extracted COVID-related tweets by HCPs, resulting in a detailed picture of clinical discourse about the disease. We explore the behavior of the proposed pipeline by comparing it to a version that ablates the concept extraction step, producing relevance estimates that highlight different characteristics of the data. Finally, we simulate the use of our technique during the early stages of a pandemic by analyzing time-limited subsets of the tweet dataset. Our results suggest this method’s potential to efficiently surface useful information to a clinical audience without significant manual analysis, potentially before such information is announced in more formal channels.

## 2 Methods

**Figure 1:**
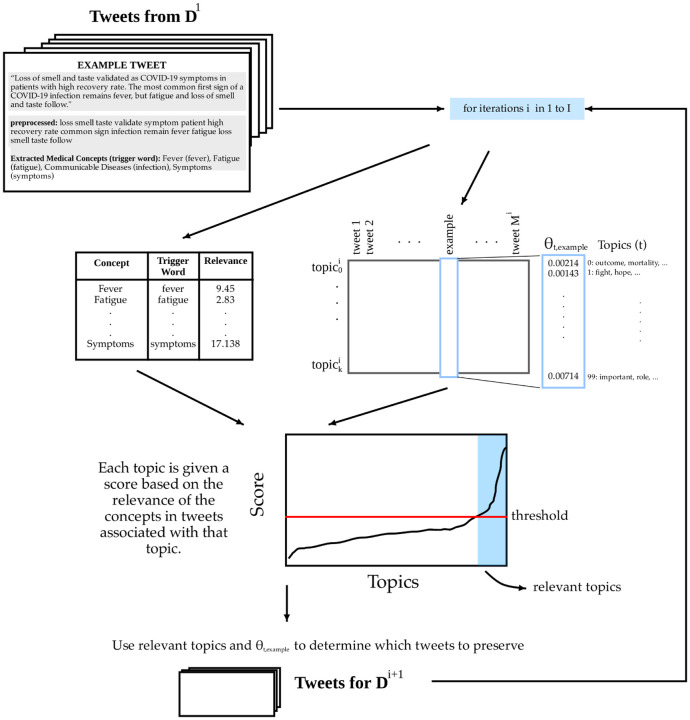
Flow chart of the iterative relevance filtering process.

In order to surface clinically relevant information in a highly noisy corpus, we develop a method based on two fundamental subroutines: topic modeling using LDA, and relevance filtering based on clinical concepts. Given an initial corpus of documents (tweets), denoted **D**^(0)^, we first apply an author-based heuristic (Section 2.1) to obtain a dataset **D**^(1)^ that has a considerably higher prior likelihood for clinical relevance. Then, we use **D**^(1)^ to produce a series of filtered subsets **D**^(*i*)^ by finding topics, computing a relevance score for each topic, and removing documents containing clinically irrelevant topics. The result of this iterative process (Section 2.3) is a set of highly relevant tweets as well as an interpretable yet granular topic model. A flowchart of this process is shown in Figure 1.

### 2.1 Heuristic author selection

To generate **D**^(1)^, we opt to only consider documents by authors who self-identify as HCPs. Social media norms suggest that it should be relatively straightforward to design a highly sensitive classifier for HCPs: audiences typically rely on author information to determine credibility on Twitter [20], so users posting about medical topics are incentivized to display their credentials. Thus, we filter **D**^(0)^ for users whose name, handle, or bio contains any of 27 medical titles, professions, or keywords (for example, “MD”, “Dr”, “epidemiolog*”, “public health”). Note that some authors use credentials falsely or in jest, and many credentialed authors post irrelevant content; these users’ documents intentionally pass the heuristic selection, and will be removed in the subsequent relevance filtering process.

### 2.2 Preprocessing and clinical concept extraction

For each document in the HCP-authored set **D**^(1)^, we preprocess the text using standard natural language processing (NLP) routines such as removing contractions, punctuation, HTML tags, and emoji. We lemmatize each word using NLTK’s WordNet lemmatizer [3], and remove stopwords and any query terms that were used to generate the original dataset (in our case study data [2], these were words explicitly referring to the coronavirus).

In addition, we extract clinical concepts from each document using MetaMap18, a tool that uses symbolic NLP to identify UMLS Metathesaurus medical concepts^1^. For a given piece of clinical text, MetaMap outputs a series of “mentions,” or occurrences of a concept defined by a unique identifier (CUI), a preferred name from UMLS, one or more semantic types (e.g. disease/syndrome or clinical finding), and trigger words (a set of words that triggered the concept match). MetaMap also outputs a relevance score, but this was not used in our protocol because it correlated poorly with our desired criteria on Twitter data.

### 2.3 Iterative relevance filtering

We perform the following iterative procedure to produce corpus **D**^(*i*+1)^ given **D**^(*i*)^ and **D**^(*i*−1)^ (for *i* ≥ 1).

#### 2.3.1 Generate topics

Using the MALLET implementation of LDA [18], we generate a topic model over the *M*^(*i*)^ documents in **D**^(*i*)^, with *k* topics (we found that *k* = 100 provides a good balance of detail and summarizability). This results in a *k* × *M*^(*i*)^ matrix *θ*^(*i*)^ that encodes topic probabilities: in particular, the value at $\theta_{t,m}^{\left(i\right)}$ is the probability that a word in document *m* was sampled from topic *t*. As described above, each document is also annotated with a certain number of concepts, which we will denote *C*(*m*).

#### 2.3.2 Score concepts

Concepts are scored by estimating the clinical relevance of each concept given its trigger word. More formally, we define relevance as a relationship between two corpora *A* and *B* where *A* ∈ *B*:

(1) $$\text{Rel}(c;A,B)=\frac{f_{A}(c)/|A|+\epsilon }{\left(f_{B}(c)-f_{A}(c)\right)/(|B|-|A|)+\epsilon }$$

where *f*_*A*_(*c*) and *f*_*B*_(*c*) are the number of occurrences of trigger word *c* in corpora *A* and *B* respectively, |*A*| and |*B*| represent the number of documents in each corpus, and *ϵ* is a small number to prevent division by zero.

Intuitively, Eqn. 1 measures how frequently concepts appear in preserved documents (*A*) as compared to discarded documents (*B* \ *A*). This formulation follows naturally from the need for an appropriate reference set, especially in the absence of labeled data. A simpler approach might be to rank concepts by inverse English word frequency, but this tends to artificially elevate alphanumeric codes (often falsely annotated by MetaMap as genes) and suppress everyday words with medical definitions (e.g. “mask,” “vent”). The iterative nature of our protocol affords us the most direct comparison possible, i.e. documents from the same data source that are known to be irrelevant.

The choice of reference set *B* is flexible: it could be held constant, or it could be set to the previously-generated corpus **D**^(*i*−1)^. Empirically, we found that a hybrid of these two approaches results in the most meaningful scores:

(2) $${\text{Rel}}^{\left(i\right)}(c)=\{\begin{array}{c} \text{Rel}\left(c;D^{\left(1\right)},D^{\left(0\right)}\right)\;\text{if }i=1\\ \text{Rel}\left(c;D^{\left(i\right)},D^{\left(1\right)}\right)\;\text{if }i>1 \end{array}$$

In other words, the relevance scores are initialized using the unfiltered set **D**^(0)^ as reference, comparing HCP to non-HCP texts. In subsequent sections, **D**^(1)^ serves as a better baseline because of its drastically improved signal-to-noise ratio compared to **D**^(0)^. We then hold **D**^(1)^ as a fixed baseline, which helps to stabilize the relevance scores over multiple iterations, and avoids honing in on a particular topic at the expense of others.

We also apply this relevance metric as a pre-filter on the MetaMap concepts: any concepts with Rel(*c*; **D**^(1)^, **D**^(0)^) < 1 (the concept occurred less frequently in doctor tweets than non-doctor tweets) are removed. This helps mitigate the signal-to-noise ratio from the very beginning, which clarifies the results of the next step.

#### 2.3.3 Score topics

Each topic is given a score based on the relevance of the concepts in tweets that are associated with it. Given the document-topic probability matrix *θ*^(*i*)^, the score of topic *t* is

(3) $${\text{Score}}^{\left(i\right)}(t)=\frac{1}{\sum_{m=1}^{M^{\left(i\right)}}\theta_{t,m}^{\left(i\right)}}\sum_{m=1}^{M^{\left(i\right)}}\theta_{t,m}^{\left(i\right)}\sum_{c\in C(m)}Rel^{\left(i\right)}(c)$$

This favors topics for which the documents drawn most heavily from the topic also contain highly relevant concepts. Note that we do not directly test for concept relevance among the topic words *β*^(*i*)^; this allows for clinically-relevant words that are *not* annotated by MetaMap (e.g. too new to appear in UMLS) to weigh heavily in topics without penalty.

The topic scores often follow an elbow-shaped curve when plotted in sorted order (see Fig. 1). We designate topics as “relevant” by choosing a threshold *τ* ∈ [0, 1] and retaining topics that satisfy

(4) $${\text{ Score }}^{\left(i\right)}(t)\geq \left(S_{\text{max}}^{\left(i\right)}-S_{\text{min}}^{\left(i\right)}\right)\cdot \tau +S_{\text{min}}^{\left(i\right)}$$

where $S_{\text{max}}^{\left(i\right)}$ and $S_{\text{min}}^{\left(i\right)}$ are the maximal and minimal topic scores, respectively. We denote this set of relevant topics *R*^(*i*)^, of size *r*^(*i*)^. We chose a threshold of *τ* = 0.25 throughout; for new datasets, *τ* could easily be set through an initial topic exploration and inspection of the resulting topics.

Notably, this thresholding scheme allows for variation in how many topics are selected: as the algorithm progresses, the number of topics retained increases with the prevalence of relevant content. The number of topics preserved in each iteration also indicates when the filtering is roughly complete.

#### 2.3.4 Filter documents

Finally, we select the documents that are highly associated with relevant topics. We generate the next corpus **D**^(*i*+1)^ by simply choosing tweets in which the probability of sampling from a relevant topic is greater than a uniform probability over topics, i.e. if $\sum_{t\in R^{\left(i\right)}}\theta_{t,m}^{\left(i\right)}\geq r^{\left(i\right)}/k$. At this point, the filtering process can be terminated if *r*^(*i*)^ is sufficiently high, or the newly-filtered corpus can be passed on to another iteration of topic modeling and relevance filtering.

## 3 Results

First, we describe our COVID-19 tweet dataset, which forms a case study for the use of our method. Then we present the results of the method on this data (Sec. 3.2), a validation of the use of concept extraction (Sec. 3.3), and a proof-of-concept for analysis on time-limited datasets (Sec. 3.4).

### 3.1 COVID-19 tweets underscore the need for relevance filtering

We illustrate the use of our method on a publicly-available COVID-19 Twitter dataset [2], comprising over 420 million tweets (as of June 21, 2020) that contain coronavirus-related keywords such as coronavirus, 2019nCoV, and covid19. Using a pre-filtered set with non-English tweets and retweets removed, we retrieved 52.9 million tweets posted between January 8 and June 21, 2020 using the twarc command line tool. Notably, many HCP-authored tweets were part of longer “threads,” or sequences of tweets by the same author, that were not fully covered by **D**^(0)^. We decided to expand the dataset in **D**^(1)^ by including the complete threads, because the missing tweets often appeared to contain useful clinical information despite not explicitly mentioning COVID keywords. The final HCP-authored tweet set contained 990,756 threads, with 1,078,830 total tweets.

Even after this initial filtering step, the tweets varied dramatically in relevance, as illustrated in Table 1. Some tweets introduce useful information, such as tweet (a) in the table. Many others contain no medical insights (tweet (b)). More subtly, however, tweet (c) contains clinical terms, but does not introduce novel information. Because of the preponderance of tweets like (b) and (c), when we attempted a large topic model (*k* = 1000) without relevance filtering, the clinically-relevant topics were vastly outnumbered by irrelevant ones. This validated the need for filtering out tweets with spurious relevance and preferentially surfacing tweets like (a).

### 3.2 Relevance filtering produces high-quality clinical topics

We performed three rounds of filtering on the HCP-authored dataset, resulting in a dataset of 107,794 tweets. Roughly 38–40 topics were selected as relevant in each iteration, but 85 topics would have been selected to proceed to the fourth iteration, indicating that most of the irrelevant data had been filtered out by this point.

**Figure 2:**
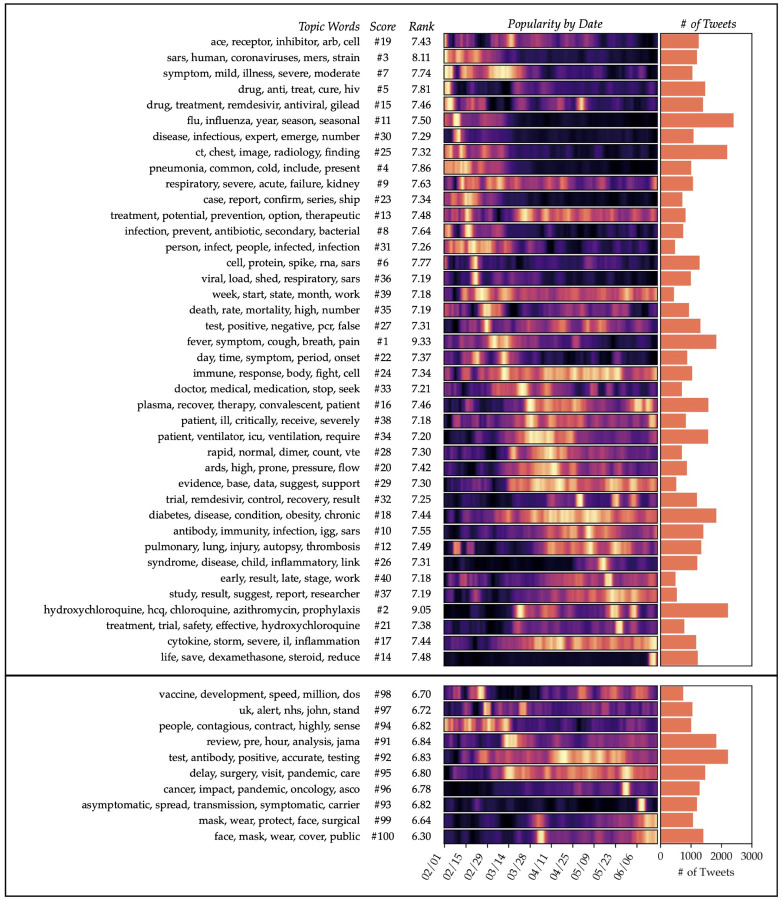
Topics generated after three iterations of relevance filtering (i.e. from **D**^(4)^). The upper section shows the top 40 highest-scoring topics, while the lower section shows the 10 lowest-scoring topics. Both sections are sorted vertically in order of the date of maximum intensity. The heat map colors indicate the popularity of each topic per day, with yellow representing the peak of popularity for the topic.

The topics extracted from the third level of filtering (**D**^(4)^), shown in Fig. 2, demonstrate clear clinical relevance. For example, several topics describe clinical presentations of COVID-19, ranging from the most common symptoms (topic #1, fever and cough) to rarer manifestations that received buzz among the medical community (#28, venous thromboembolism; #26, Kawasaki syndrome). Some topics describe the underlying physiological conditions that lead to these symptoms, such as #17 (cytokine storms) and #19 (the ACE2 receptor, which is implicated in viral entry to the cell). Still other topics discuss new and emerging treatments, such as #16 (convalescent plasma therapy), #2 (hydroxychloroquine and azithromycin), #14 (dexamethasone) and #15 (remdesivir).

Meanwhile, the less relevant topics in the third-iteration model tend to deal with the boilerplate aspects of clinical tweets, such as #91 (announcing study pre-prints), as well as topics adjacent to COVID-19 but not directly applicable (#95 and #96 refer to secondary effects of the pandemic on healthcare). Interestingly, the topics considered least relevant (#99 and #100) are the ones that deal with surgical and everyday masks, respectively. These tweets are likely considered irrelevant because they often touch on the U.S. politicization of mask wearing, or address the topic in a public-safety announcement format, such as “COVID is still spreading out there. Wear your mask. Practice social distancing.” Irrelevant topics will always be present in some frequency because they co-occur in the same documents as relevant topics, but by presenting the topics in ranked order, the model still facilitates interpretation over an unsorted topic list.

### 3.3 Concept extraction improves focus on clinical terms

To validate our use of MetaMap concept annotations when calculating relevance, we ran a version of our iterative filtering routine that entirely omitted clinical concepts. In other words, the relevance of a topic (Eqn. 3) was computed not as the sum of relevances over *concepts*, but instead over *all* words in each tweet (excluding stopwords and words explicitly mentioning the coronavirus). This comparison therefore reflects the marginal benefit of directing the relevance filtering toward words already known to be clinically relevant (subject to MetaMap error).

First, we compared the words that were predicted to be most and least relevant by each method, shown in Table 2. Relevance is calculated using Eqn. 2 for all unigrams, bigrams, and trigrams; this therefore also serves as a measure of what phrases are most “enriched” in the relevant tweet set compared to the irrelevant tweet set. The first column, which shows the enrichment of **D**^(1)^ relative to **D**^(0)^, suggests that heuristic author filtering alone establishes a fairly strong baseline, independent of the use of clinical concepts. Still, after three rounds of relevance filtering, the top phrases in **D**^(4)^ show a marked improvement over **D**^(1)^. Using concepts in the filtering process led to an emphasis on *clinical* content (“lung,” “respiratory,” “ards”), while using all words resulted in a greater proportion of *epidemiological* content (“mortality,” “asymptomatic,” “growth rate”). This could be because of a natural bias in UMLS toward concepts used in a hospital setting, or because epidemiological topics received the most sustained attention while areas of medical interest shifted. Therefore, while using concepts better suited our objective of extracting clinically-relevant information in this case, omitting the MetaMap step in our pipeline could still result in useful filtering for a different application.

Next, to ensure our method was accurately discriminating relevant and irrelevant tweets, we examined the effect of filtering on several topics known to be either relevant or irrelevant to the pandemic. With the guidance of a clinician, 12 categories and associated keywords were chosen such that if *any two* of the keywords were present in a tweet, its relevance could be gauged with relative certainty. For example, any tweet containing both “anosmia” and “dysgeusia” should most likely never be eliminated. Similarly, any tweet containing the names of political leaders is most likely irrelevant. A total of 26,851 tweets were used for this analysis.

**Figure 3:**
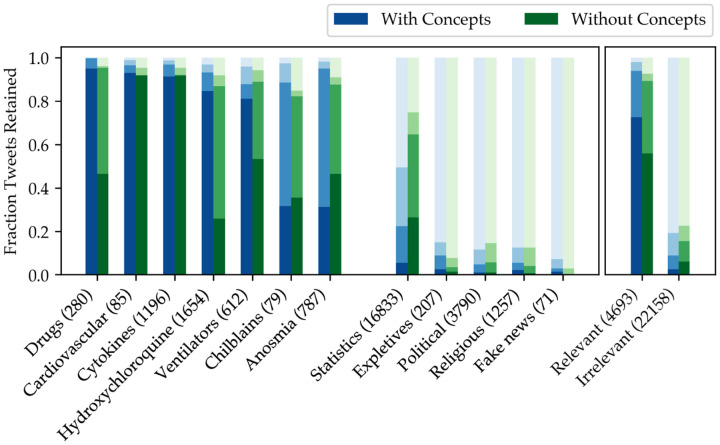
Fraction of tweets preserved by two different models (with and without using clinical concepts for relevance filtering) for each of 12 pre-defined tweet categories, including clinically-relevant subjects (first seven bars) and irrelevant ones (next five). The progressively darker bars represent successive stages of filtering. The parenthesized number indicates the number of tweets that fell into each category. The total fractions for the irrelevant and irrelevant categories are shown in the right panel.

Figure 3 shows the proportion of tweets in each category that were retained by the filtering process in each iteration, with the darkest bar representing the proportion kept after three rounds. Based on the aggregation of categories to the right of the figure, using concept extraction leads to an increase in relevant tweets preserved (72.5% with concepts, 55.8% without), and a decrease in irrelevant tweets (2.5% with concepts, 5.9% without). Concept filtering performed especially well on the “Drugs” category, likely because the drug mentions were consistently annotated and scored well for relevance. On the other hand, concept-based filtering performed poorly by this test on “Chilblains” and “Anosmia,” both of which are referenced using common words (“toe,” “smell”) that were ranked lower by the concept relevance metric than other clinical terms. While the categories used here are imperfect representations of relevance and irrelevance, concept extraction does provide an advantage in preserving the correct sets of tweets.

### 3.4 Automatic relevance filtering enables real-time trend analysis

**Figure 4:**
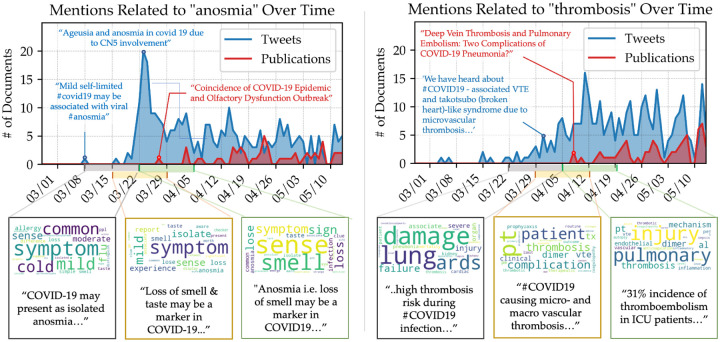
Analysis of two surfaced clinical concepts: “anosmia” and “thrombosis.” The histograms are labeled with examples of mentions in tweets and publications. Topics containing the concept are shown as word clouds; the size of the word correlates to its weight in the topic. A tweet containing the topic is also shown.

If this method were applied in the early stages of a pandemic, it would be important to identify emerging topics within a matter of days. To see if our method could recover nascent topics in this time-limited manner, we split our data into 2-week subsets overlapped by 1 week, then applied two rounds of relevance filtering to each subset. Figure 4 shows two case studies of concepts that surfaced in these time-limited topic models: anosmia (or lack of smell) and thrombosis (intravenous blood clots). For a comparison to the academic literature, the upper plot shows when these clinical concepts first appeared in CORD-19, a dataset of COVID-related publications [26].

For topics related to anosmia, the week-to-week microtrends are clearly visible in the time-limited topic models. While anosmia is now known to be a symptom of COVID-19, in March 2020 the connection between anosmia and COVID-19 was not yet well established. In topics that contain anosmia-related keywords from three consecutive time windows (Fig. 4, lower left), words describing loss of smell grow progressively more significant, which tracks with the increasing number of tweets about anosmia during this time period.

Because some emerging conditions contain very low tweet counts before they enter the literature, they may not always be isolated as individual topics. For example, words related to thrombosis (Fig. 4, lower right) never become a topic in their own right in the time-limited models, although they were present alongside related conditions. This underscores the importance of looking at the tweets associated with topics of interest, which do contain early insights about thrombosis.

We also found that preliminary clinical information was surfaced in our topic models *before* its mention in academic publications. For example, the topic model from March 15–28 surfaced several tweets mentioning links between anosmia and COVID (e.g. “Too many reports of loss of smell, taste or both in people with coronavirus for this to be a coincidence.”, March 23, 2020). Meanwhile, the first academic article describing this phenomenon that appears in CORD-19 was only published at the end of this interval (March 27). As seen in Figure 4 (top), clinical conversations about anosmia and thrombosis were transpiring on Twitter at sufficient levels to manifest in the filtered topic models before academic publications appeared. This suggests our method can be useful in rapidly-changing situations as it can surface clinically relevant information as they are being discovered and discussed.

## 4 Discussion

This study provides a new look at social media for rapidly-evolving public health situations, and develops a strategy for extracting granular clinical topics without manual labeling. As with all social media applications, a key challenge in information extraction from Twitter is the signal-to-noise ratio: our filtering process reduced the initial COVID dataset to about 0.2% of its original size. Furthermore, because of the pandemic’s impact on everyday life, many tweets can contain medical terminology without necessarily imparting clinically relevant information. Our proposed technique resolves these issues by applying topic modeling and concept extraction in tandem, resulting in progressively better estimates of clinical relevance.

Traditional topic modeling is a valuable first step in understanding the contents of any textual dataset, and we observed that our topic models often naturally grouped together relevant and irrelevant information. However, the larger and more granular our models became, the harder it was to find relevant topics without a ranking strategy (which led to the development of our method). On the other hand, MetaMap concept extraction can identify relevant phrases regardless of their frequency, a useful complement to statistical NLP methods. Using these annotations to rank the topic models allows one to quickly discard irrelevant categories, improving interpretability considerably for larger models. Concept extraction therefore approximates a physician’s prior belief of which topics should be considered *relevant*, while the topic-document mapping approximates which documents should be considered *related*.

An iterative approach is key to enabling topic modeling and concept extraction to refine and validate each other’s results. For example, in early iterations, we observed many tweets that were associated with relevant topics because they contained clinical terminology, but that were not themselves written in a medical “frame.” As these tweets were carried forward into subsequent levels, however, topics became more relevant overall, and tweets that used clinical terminology in a non-medical context became sequestered into their own topics.

Analyzing topic models from HCP-authored tweets opens opportunities for medical professionals to learn from each other and initiate new lines of research. When clinical indicators are highlighted by our method as they emerge, as suggested by the two-week topic models, HCPs in regions with rising cases could browse topics and tweets from previous epicenters to better understand what to expect. Clinicians who were not part of the original conversation but who have observed the emerging symptom could then contribute knowledge to ongoing research, enabling wider information sharing and even expanding access to clinical trials. Although the analysis relies to some degree on the topics already being relatively prevalent on social media, it can still accelerate awareness of these findings outside of the circles in which they are shared.

As with any study involving Twitter data, our analysis is subject to the biases and caveats inherent in social media. The dataset collected here only consisted of English tweets, but it could be more helpful to apply the technique across languages and capture a truly global conversation, perhaps by integrating a multilingual topic model [6]. Even among English-speaking HCPs, this dataset is hardly a representative sample of all medical opinion on the pandemic, since the demographics of physicians on Twitter are inevitably skewed by the demographics of Twitter itself. Finally, the rapid oscillations of interest in the generated topics suggest that HCPs, like other Twitter users, gravitate toward the most exciting stories at any given time. The degree of intensity of a topic, therefore, will always be an imperfect proxy for true clinical importance.

## 5 Conclusions

We present a method that combines topic modeling and clinical concept extraction to surface clinically relevant information from a noisy text dataset. Both topic modeling and concept annotation have their limitations on this type of data; nevertheless, we have shown that iterating between the two techniques can overcome these weaknesses. This is especially important in emergent situations such as COVID-19, where unconventional data sources and unsupervised information extraction are often the only option for rapid analysis. Our pipeline can be applied without modification to social media data on other medical topics, and the iterative filtering technique could be a helpful augmentation to topic models used for preliminary explorations of text data.

In seeking to extract clinical information from social media data, this study addresses a problem space that has not yet been addressed to our knowledge. Indeed, in normal circumstances the rapid fluctuations of social media trends are antithetical to robust, reliable official guidance. As demonstrated by our analysis, however, HCP-authored tweets about COVID-19 can present a surprisingly bountiful window into the epicenters of a pandemic, and we hope that our method enables future research to gain deeper insights from the physician population on social media. By automatically bringing clinical tweets out of the limited audiences among whom they are shared, newly-emerging clinical observations can be disseminated quickly and clinicians everywhere can contribute to shared knowledge. Just as Twitter data reflects the rapidly-changing world, this method could enable the flexible, real-time analysis that a pandemic demands.

## Figures and Tables

**Table 1:** Paraphrased example tweets from the HCP-authored subset of the dataset

(a) GI symptoms (anorexia, diarrhea, vomiting, or abdominal pain) may be earlier signs of novel #coronavirus, presenting before respiratory symptoms #medtwitter	(b) Feeling low, stressed, or anxious? Free programs available for those experiencing #MentalHealth challenges associated with #COVID19 or life’s other issues!	(c) We hate the drug! Take it off the market!!! 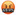 * Chloroquine lowers fever in patients with coronavirus* Cholorquine: 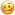

**Table 2:** Words and phrases that were designated most (upper half) and least (lower half) relevant according to Eqn. 2 (relevance values shown in parentheses). The first column indicates the initial relevance estimates computed after heuristic author filtering; the right two columns list the relevances after three rounds of filtering with and without concept annotations.

	D^(1)^	D^(4)^ with concepts	D^(4)^ without concepts
1	medtwitter *(6.33)*	cells *(20.18)*	cells *(28.71)*
2	publichealth *(4.70)*	lung *(17.67)*	mortality *(28.05)*
3	physicians *(4.14)*	hcq *(16.03)*	rate *(26.57)*
4	patients with *(3.88)*	trial (15.85)	asymptomatic (*22.21*)
5	pts (3.85)	patients with *(13.96)*	rate of *(20.66)*
6	clinical *(3.81)*	severe *(13.64)*	fatality *(18.86)*
7	physician (3.66)	respiratory *(13.63)*	mild *(18.41)*
8	icu *(3.44)*	blood *(12.97)*	growth rate *(17.24)*
9	surgery *(3.34)*	antibodies *(12.46)*	viral *(16.51)*
10	md (3.33)	ards *(12.40)*	ace2 *(16.24)*
1	f*** *(0.44)*	business *(0.18)*	trump *(0.14)*
2	s*** (0.48)	leadership *(0.20)*	business *(0.15)*
3	f***ing *(0.48)*	businesses *(0.23)*	bbc news *(0.19)*
4	petition *(0.49)*	students *(0.23)*	bbc *(0.20)*
5	the petition *(0.50)*	government *(0.23)*	president (*0.21*)
6	rt *(0.50)*	trump (0.23)	news coronavirus *(0.24)*
7	sign the petition (0.53)	crisis *(0.24)*	bbc news coronavirus *(0.25)*
8	democrats (0.53)	county *(0.24)*	the latest (0.25)
9	sign the (0.53)	pm *(0.24)*	businesses *(0.26)*
10	the petition via *(0.54)*	economy *(0.25)*	amid *(0.26)*

## Footnotes

1 In this study, we reduced computational overhead by only applying MetaMap to tweets that contain at least 4 words. Shorter tweets often contained reactions and links, which are not useful for our text analysis purposes.
